# A new TAO kinase inhibitor reduces tau phosphorylation at sites associated with neurodegeneration in human tauopathies

**DOI:** 10.1186/s40478-018-0539-8

**Published:** 2018-05-07

**Authors:** Caterina Giacomini, Chuay-Yeng Koo, Natalia Yankova, Ignatius A. Tavares, Selina Wray, Wendy Noble, Diane P. Hanger, Jonathan D. H. Morris

**Affiliations:** 1 King’s College London, School of Cancer and Pharmaceutical Sciences, New Hunt’s House, Guy’s Campus, London, SE11UL UK; 2 King’s College London, Department of Basic and Clinical Neuroscience, Maurice Wohl Clinical Neuroscience Institute, Institute of Psychiatry, Psychology & Neuroscience, 5 Cutcombe Road, London, SE59RX UK; 3 UCL Institute of Neurology, Department of Molecular Neuroscience, University College London, 1 Wakefield Street, London, WC1N1PJ UK

**Keywords:** Alzheimer, Tauopathy, Dementia, TAOK, Tau, Phosphorylation, Kinase

## Abstract

In Alzheimer’s disease (AD) and related tauopathies, the microtubule-associated protein tau is highly phosphorylated and aggregates to form neurofibrillary tangles that are characteristic of these neurodegenerative diseases. Our previous work has demonstrated that the thousand-and-one amino acid kinases (TAOKs) 1 and 2 phosphorylate tau on more than 40 residues in vitro. Here we show that TAOKs are phosphorylated and active in AD brain sections displaying mild (Braak stage II), intermediate (Braak stage IV) and advanced (Braak stage VI) tau pathology and that active TAOKs co-localise with both pre-tangle and tangle structures. TAOK activity is also enriched in pathological tau containing sarkosyl-insoluble extracts prepared from AD brain. Two new phosphorylated tau residues (T123 and T427) were identified in AD brain, which appear to be targeted specifically by TAOKs. A new small molecule TAOK inhibitor (Compound 43) reduced tau phosphorylation on T123 and T427 and also on additional pathological sites (S262/S356 and S202/T205/S208) in vitro and in cell models. The TAOK inhibitor also decreased tau phosphorylation in differentiated primary cortical neurons without affecting markers of synapse and neuron health. Notably, TAOK activity also co-localised with tangles in post-mortem frontotemporal lobar degeneration (FTLD) brain tissue. Furthermore, the TAOK inhibitor decreased tau phosphorylation in induced pluripotent stem cell derived neurons from FTLD patients, as well as cortical neurons from a transgenic mouse model of tauopathy (Tau35 mice). Our results demonstrate that abnormal TAOK activity is present at pre-tangles and tangles in tauopathies and that TAOK inhibition effectively decreases tau phosphorylation on pathological sites. Thus, TAOKs may represent a novel target to reduce or prevent tau-associated neurodegeneration in tauopathies.

**Electronic supplementary material:**

The online version of this article (10.1186/s40478-018-0539-8) contains supplementary material, which is available to authorized users.

## Introduction

Pathological aggregation of highly phosphorylated tau and the formation of neurofibrillary tangles (NFTs) in the brain provide one of the main characteristic pathological features of a subgroup of neurodegenerative disorders termed tauopathies. Alzheimer’s disease (AD) is the most prevalent tauopathy but similar tangle-like features are also displayed in frontotemporal lobar degeneration (FTLD), Pick’s disease, progressive supranuclear palsy and corticobasal degeneration [17, 19]. The presence of extracellular plaques of beta-amyloid (Aβ) provides an additional hallmark for AD. However, the emergence of tau pathology appears to correlate more closely than plaques with the progression of cognitive impairment in AD [26].

Human tau is encoded by the *MAPT* gene located on chromosome 17 and alternative splicing of exons 2, 3 and 10 produces six tau isoforms containing up to two inserts at the N terminus (0 N, 1 N or 2 N) and three or four repeated sequences (3R or 4R) at the C terminus of the protein [8, 36]. Tau is expressed predominantly in neurons and is a major microtubule-associated protein (MAP) involved in the regulation of microtubule dynamics and organisation [13, 58]. Tau splicing is developmentally regulated and only the shortest 0N3R tau isoform is expressed in the foetal brain, whereas all six tau isoforms are present in the adult central nervous system (CNS) [8, 36]. In AD and related tauopathies, aberrant tau phosphorylation results in its dissociation from microtubules and the aggregation of tau in the cytosol to form NFTs [23, 34]. The relative importance of individual or combinations of phosphorylated tau residues remains to be determined. Phosphorylation of the KXGS repeated motifs (notably S262 and S356) located in the C-terminal microtubule binding domain are involved in regulating tau microtubule-binding, as are additional phosphorylation sites flanking this region [1–3, 22].

A number of protein kinases have been shown to target tau, at least in vitro, including microtubule-affinity regulating kinase (MARK), glycogen synthase kinase 3 (GSK-3), cyclin-dependent kinase 5 (CDK5), Fyn and also extracellular signal regulated kinase 2 (ERK2), c-Jun N-terminal kinase (JNK) and p38 mitogen-activated protein kinases (MAPKs) [3, 24, 33, 40, 48, 61]. An important challenge for dementia is to identify kinases that are catalytically active, associated with tangles, and are therefore likely to contribute directly to the development of tau pathology [20]. Previously, we have reported that the thousand-and-one amino acid kinases (TAOKs, also referred to as prostate-derived sterile 20-like kinases (PSKs)) phosphorylate tau on multiple disease-associated sites and that these kinases are activated in AD brain [55]. TAOKs belong to the sterile 20 group of mammalian protein kinases, with family members including TAOKs 1–3 [9, 25, 44, 69]. TAOK1 and TAOK2 can regulate MAPKs and stimulate JNK and p38 MAPK signalling pathways [9, 25, 44, 68, 69]. TAOK1 and TAOK2 also induce apoptotic changes via activation of JNK, MAPK and caspases; however, a key feature of TAOKs is their ability to regulate microtubule dynamics and organisation [38, 43, 56, 68]. TAOK1 induces microtubule instability through its direct activation of MARK and phosphorylation of MAPs, including tau, which dissociate from microtubules, resulting in their disassembly [55–57]. TAOK2 binds to microtubules directly through its C terminus (amino acids 745–1235), leading to stabilisation of perinuclear microtubules, which contain increased acetylated α–tubulin and are resistant to nocodazole-induced depolymerisation [43].

Although very little is known about the relationship of TAOKs to neurodegenerative diseases, the TAOK2 gene is located on chromosome 16p11.2, a region that carries significant susceptibility for autism spectrum disorders and schizophrenia [41, 62]. In the developing mouse brain, TAOK2 is required for basal dendritic development and callosal axon projection in cortical neurons, where it acts downstream of the neuropilin-1 receptor for semaphorin 3A, to regulate dendrite arborisation and axon elongation through JNK [10]. Phosphorylation of TAOK1 (pT440) and TAOK2 (pT475) by mammalian sterile 20-like kinase 3 (MST3) results in myosin Va binding and subsequent localisation of these kinases to dendrites, where they regulate spine and synapse development [59]. TAOK2 also mediates post-synaptic density protein 95 (PSD95) stability and dendritic spine maturation via phosphorylation and relocation of the cytoskeletal GTPase septin 7 [65]. A recent study has also identified three missense mutations in *TAOK2* in autism spectrum disorder subjects and shown that the expression of mutated forms of TAOK2 or increased expression of wild type TAOK2 impairs dendritic spine development in primary neurons [49]**.** These studies demonstrate important functions for TAOK2 during early brain development; however, the potential roles for TAOKs in the adult brain and in the aberrant phosphorylation of tau in tauopathies have not yet been explored.

In this study, we have investigated the effects of tau phosphorylation resulting from TAOK activity and inhibition using a new small molecule inhibitor. We found that TAOK activity was present in pre-tangles and tangles in AD and FTLD brain, whereas it was only weakly detectable in control human brain. TAOKs phosphorylated tau on pathological sites in human cell lines in addition to murine and human neurons, and this was reduced by the TAOK inhibitor. Our findings implicate the TAOKs as important contributors to the development of tau pathology in AD and FTLD.

## Materials and methods

### Plasmids, antibodies and reagents

Plasmids pRK5-TAOK1, pRK5-TAOK2, pRK5-TAOK2 (K57A) and pRK5-TAOK2 (amino acids 1–349) were generated as described previously [44, 69] and subcloned into pCAGGS-IRES-EGFP or pCAGGS-IRES-Tomato vectors (gift from Dr. L. Gasparini, Italian Institute of Technology, Genoa, Italy). PLenti-lox3.7 vectors expressing TAOK2-shRNA or non-targeting-shRNA and enhanced green fluorescent protein (EGFP) using an alternative promoter were a gift from Dr. S. Ultanir (Crick Institute, London, UK). Tau-pT123, tau-pT427 and TAOK-pS181 affinity-purified rabbit antibodies and peptides were produced by Eurogentec. Mouse tau-pS262/S356 antibody (12E8) was a gift from Dr. P. Seubert (Prothena, USA). Commercially available mouse monoclonal antibodies were obtained to detect TAOK1 (BD), tau-pS202/T205/S208 (AT8, Thermo Fisher Scientific), total tau (Tau5, Thermo Fisher Scientific), synaptophysin (SP15, Enzo), Myc (9E10, Sigma-Aldrich) and glyceraldehyde 3-phosphate dehydrogenase (GAPDH) (Merck Millipore). Rabbit antibodies were obtained to recognise TAOK1 and TAOK2 (Proteintech), BIII Tubulin (Sigma-Aldrich), total tau (Dako), synapsin 1 (Merck Millipore) and active cleaved caspase III (R&D Systems). GST-TAOK1 (amino acids 1–314) and GST-TAOK2 (amino acids1–314) were obtained from SignalChem. Reagents and chemicals were purchased from Sigma-Aldrich and cell culture reagents were obtained from Thermo Fisher Scientific, unless otherwise stated. Compound 43 was made by Evotec as described previously [29].

### Immunostaining of human brain tissue

Clinically and pathologically confirmed post-mortem human AD, FTLD and control brains (Additional file 1: Table S1) were obtained from the MRC London Neurodegenerative Disease Brain Bank (King’s College London). Tissue sections (7 μm) were cut from formalin-fixed paraffin-embedded blocks of human brain. Sections were deparaffinised and endogenous peroxidase activity inhibited by incubating samples in 3% (*v*/v) hydrogen peroxide (30 min) followed by antigen retrieval using enhanced microwaving in 10 mM sodium citrate buffer (20 min) (pH 6.0). Sections were blocked for 1 h in 10% (v/v) goat serum before incubation overnight with TAOK-pS181, tau-pT123 or tau-pT427 antibodies (4 °C). Sections were incubated with goat anti-rabbit-biotinylated secondary antibodies (45 min, Dako) and developed using the VECTASTAIN Elite ABC kit (Vector Laboratories) and 0.5 mg/ml 3, 3′-diaminobenzidine chromogen (Vector Laboratories). All sections were counterstained with hematoxylin. For double immunofluorescence labelling, sections were pre-treated as described above, and incubated overnight with phospho-tau antibodies (4 °C) followed by secondary goat anti-rabbit 568 or goat anti-mouse 488 Alexa Fluor antibodies (Thermo Fisher Scientific). Autofluorescence was quenched by Sudan black (0.1% Sudan black in 70% ethanol) and images were acquired using a CSU-X1 inverted spinning-disk confocal microscope (Nikon) equipped with EM-CCD camera (Andor iXon3) and a 60×/1.40 NA oil objective (Nikon).

### Extraction of sarkosyl-insoluble tau

Sarkosyl extraction of tau from brain tissue was performed as described previously [30]. Briefly, brain tissues were homogenised in 50 mM Tris buffered saline (TBS, pH 7.4) containing 2 mM EGTA, 1 mM Na_3_VO_4_, 10 mM NaF and 1 mM phenylmethylsulfonyl fluoride. Samples were centrifuged at 20,000 *g* for 20 min (4 °C) and sarkosyl (10% *v*/v) was added to the resultant supernatant to give a final concentration of 1% (*v*/v). Samples were mixed for 30 min at ambient temperature, then centrifuged at 100,000 *g* for 1 h at ambient temperature. The supernatant was collected and the pellet washed twice with 1% (v/v) sarkosyl before solubilisation in 2× sodium dodecyl sulphate (SDS) sample buffer (National Diagnostics). The three fractions produced comprised (i) low speed supernatant, (ii) sarkosyl-soluble tau, and (iii) sarkosyl-insoluble tau. Proteins in the samples were resolved by SDS gel electrophoresis (SDS-PAGE) and processed for immunoblotting.

### In vitro kinase assays

3 μg human recombinant tau (2N4R isoform, Sigma-Aldrich) and 30 ng purified glutathione S-transferase (GST)-TAOK1 (1–314) or GST-TAOK2 (1–314) were incubated in kinase buffer (20 mM MgCl_2_, 2 mM MnCl_2_, 3 mM ATP and 30 mM Tris, pH 7.4) for 6 h at 30 °C. Reactions were terminated in 5× Laemmli gel sample buffer (10% SDS, 50% glycerol, 0.05% bromophenol blue, 350 mM dithiothreitol and 1 M Tris, pH 6.8). Samples were resolved by SDS-PAGE and protein phosphorylation was analysed on immunoblots probed with appropriate antibodies.

### Culture of HEK293T cells and primary cortical neurons

Human embryonic kidney (HEK) 293 T cells were cultured in Dulbecco’s Modified Eagle’s Medium (DMEM, Sigma-Aldrich), supplemented with 10% (*v*/v) foetal calf serum (FCS, Thermo Fisher Scientific) and antibiotics, at 37 °C in an atmosphere of 5% CO_2_. Cells were transfected using Lipofectamine 2000 (Thermo Fisher Scientific) according to the manufacturer’s instructions. Cultured HEK293T cells were incubated with TAOK inhibitor compound 43 (Cp 43), or equivalent concentrations of vehicle dimethyl sulfoxide (DMSO and to a maximum of 0.3%), for 24 h. Treated cells were collected, lysed and analysed on western blots. Rat primary cortical neurons were prepared from embryonic (E) day 18.5 brains as described previously [16] and neurons were seeded onto poly-L-lysine–coated coverslips or 6 well plates at 3–8 × 10^4^ cells per coverslip (18 mm) or at 5 × 10^5^ cells per well. Neurons in neurobasal medium containing B-27, GlutaMAX and antibiotics (Thermo Fisher Scientific) were cultured at 37 °C, in an atmosphere of 5% CO_2_. Primary cortical neurons were prepared from Tau35 transgenic mice [4] and cultured as described above. Neurons were treated with Cp 43 or vehicle for the indicated times and concentrations, adding fresh inhibitor daily for longer time points. Cells were either lysed for analysis on western blots or stained for immunofluorescence analysis. Neurons transfected with Lipofectamine 2000 were fixed and stained for immunofluorescence analysis. All methods were carried out in accordance with the UK Animals (Scientific Procedures) Act 1986.

### Culture of induced pluripotent stem cell-derived neurons

Induced pluripotent stem cells (iPSCs) from control and FTLD patients carrying the MAPT 10 + 16 mutation were prepared as described previously [53]. IPSCs were differentiated into cortical neurons using dual SMAD inhibition followed by in vitro neurogenesis, as described previously [52, 53]. After culture for 28–30 days in vitro (DIV), when a substantial amount of neurogenesis had occurred, cells were plated onto poly-ornithine and laminin coated plates (Sigma-Aldrich) or on eight-well chamber slides for immunofluorescence (Ibidi) and fed every two days with neuronal maintenance media (N-2 medium and B-27 medium, ratio 1:1). N-2 medium consists of DMEM/F-12 GlutaMAX containing 1 x N-2, 5 μg/ml insulin, 1 mM L-glutamine, 100 μM non-essential amino acids, 100 units M2-mercaptoethanol, 50 U/ml penicillin and 50 mg/ml streptomycin. B-27 medium consists of Neurobasal medium containing 1 x B-27, 20 mM L-glutamine, 50 U/ml penicillin and 50 mg/ml streptomycin (Thermo Fisher Scientific). Neurons were treated with Cp 43 or vehicle, and cultures were either lysed for immunoblotting or stained for immunofluorescence analysis at 60 DIV.

### Immunofluorescence

Primary cortical neurons were fixed with 4% (*w*/*v*) paraformaldehyde (PFA) in phosphate-buffered saline (PBS, pH 7.4) for 15 min, permeabilised with 0.2% (w/v) Triton X-100 in PBS for 5 min, and blocked with 10% (*v*/v) goat serum and 0.25% (w/v) Triton X-100 in PBS for 30 min. Neurons were incubated sequentially overnight at 4 °C with appropriate primary antibodies followed by Alexa Fluor 488-, 568- or 647-conjugated secondary antibodies (2 h). Coverslips were mounted using Prolong Gold (Thermo Fisher Scientific) containing 4′, 6′-diamidino-2-phenylindole (DAPI) to label nuclei. To quantify tau phosphorylation on threonine 427 (T427) in neurons transfected with TAOK2 and treated with Cp 43 or vehicle, z-stack confocal images were acquired using fixed laser and scanning settings. Mean immunofluorescence intensity was measured using ImageJ software [50]. Results are expressed as the amount of tau-pT427/tau5 (total tau) immunofluorescence intensity.

### Cytotoxicity assays

Rat primary neurons were treated with Cp 43 (6 h) and cytotoxicity assessed by measuring LDH release into the culture medium using a Cytotox 96 assay kit (Promega, Madison, WI, USA), according to the manufacturer’s instructions. Optical density was measured at 492 nm and LDH release from neuronal cultures was expressed as a percentage of total LDH in each sample. Cytotoxicity was also assessed by quantifying through immunofluorescence staining the number of active caspase 3-positive cells following treatment with Cp 43 (72 h). Quantification was carried out on blind-coded sample images using ImageJ software.

### Western blotting

HEK293T cells, primary and iPSC derived neurons were lysed in RIPA buffer (1% NP-40, 0.1% SDS, 150 mM NaCl and 50 mM Tris pH 7.5) supplemented with protease and phosphatase inhibitors (Sigma-Aldrich). Lysed cells were sonicated for 5 min and clarified by centrifugation at 10,000 g for 10 min at ambient temperature. Protein concentrations were determined using a bicinchoninic acid assay (Thermo Fisher Scientific). Equal amounts of protein (15–30 μg) in 5× Laemmli sample buffer were resolved by SDS-PAGE and transferred to 0.2 μm nitrocellulose membranes (GE Healthcare). Membranes were blocked in 5% (*w*/*v*) skimmed milk in buffer (150 mM NaCl, 0.1% Tween 20 and 10 mM Tris, pH 7.5), incubated with appropriate primary antibodies overnight at 4 °C, followed by horse radish peroxidase (HRP)-conjugated secondary antibodies (Dako) for 1 h at ambient temperature. Protein bands were visualised using SuperSignal West Pico Chemiluminescent Substrate (Thermo Fisher Scientific) and Hyperfilm ECL (GE Healthcare) and densitometric analysis was carried out using ImageJ software.

### Statistical analysis

Statistical analysis of data was performed using Student’s *t*-test for two groups or ANOVA followed by the Holm-Sidak post hoc test in case of multiple comparisons (Sigma Plot 13.0 Software). Differences between groups were considered statistically significant when *p* < 0.05. Results are reported as mean values ± SEM, except when specified otherwise.

## Results

### Active TAOK-pS181 associates with tau pathology in AD and FTLD brain

**Fig. 1:**
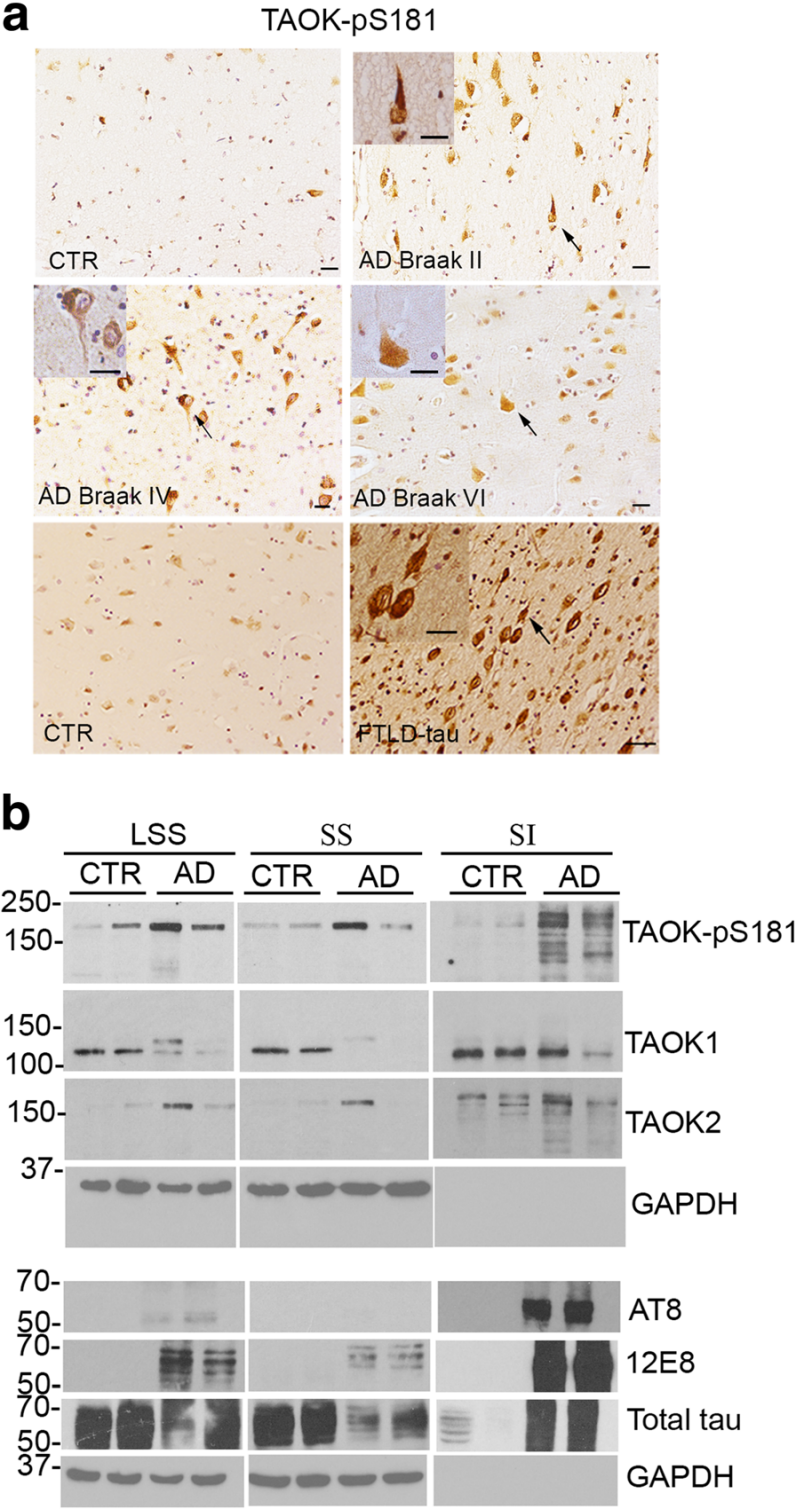
TAOK-pS181 associated with tau pathology in AD and FTLD-tau brain. **a** Formalin-fixed sections from the entorhinal cortex of AD (Braak stages II, IV and VI), FTLD-tau or control brains were immunostained with antibody to detect phosphorylated and active TAOKs (TAOK-pS181). Strong positive antibody immunoreactivity was observed on NFTs in AD brain sections from early and late disease stages and in FTLD brain sections (arrows indicate magnified NFTs). **b** AD (Braak VI) or control (CTR) entorhinal cortex brain samples were extracted with sarkosyl and low speed supernatants (LSS) or sarkosyl-soluble (SS) or sarkosyl-insoluble (SI, insoluble tau) fractions immunoblotted with antibodies to detect TAOK1, TAOK2, TAOK-pS181 and GAPDH or tau-pS262/S356 (12E8), tau-pS202/T205/S208 (AT8), total tau and GAPDH. Representative images and immunoblots are shown. Scale bar = 20 μm

Previously, we have used a phospho-antibody (TAOK-pS181) to detect forms of TAOK1 and TAOK2 that are catalytically active and phosphorylated on serine residue 181 (S181) as part of their conformational activation [63, 66, 68]. To investigate the contribution of TAOKs to the development of tau pathology and disease progression in AD, serial sections were prepared from the entorhinal cortex of AD brain displaying mild (Braak stage II, *n* = 3), moderate (Braak stage IV, *n* = 3) or severe (Braak stage VI, *n* = 4) pathology alongside control brain (*n* = 4) (Additional file 1: Table S1) [5]. Sections (× 12 per brain sample) were immunostained with the TAOK-pS181 antibody, and strong positive immunoreactivity was observed in tangles and pre-tangle structures in all sections from AD brains with mild, moderate or severe Braak stages (Fig. 1a, Braak stages II, IV, VI). In contrast, no tangle-like structures were apparent with TAOK-pS181 staining in control brain (Fig. 1a). The accumulation of highly phosphorylated tau aggregates at tangles not only provides a key feature for AD but is also characteristic for a number of related tauopathies, including frontotemporal lobar degeneration with tau-immunoreactive inclusions (FTLD-tau) [17]. Consequently, entorhinal cortex sections (× 12 per brain sample) from two individuals with FTLD-tau were immunostained with the TAOK-pS181 antibody and positive immunoreactivity was detected in tangle-like structures in all sections (Fig. 1a, Additional file 1: Table S1). These results imply that TAOK activity may also contribute to tau pathology and tangle formation in additional tauopathies such as FTLD-tau.

The association between TAOK expression and activity and tau pathology was investigated further by preparing extracts from the entorhinal cortex of two Braak stage VI AD and two control brains. Low speed supernatants (LSS), sarkosyl-soluble (SS) and sarkosyl-insoluble (SI, insoluble tau) brain fractions were analysed on western blots probed with antibodies recognising active TAOKs, TAOK1, TAOK2 or GAPDH (Fig. 1b). TAOK1 (~ 110 kDa) and TAOK2 (~ 175 kDa) displayed apparent molecular weights that were similar to those reported previously for TAOK1 and TAOK2 [25, 44] and their expression levels in AD and control samples were comparable in the SI fraction (Fig. 1b). Phosphorylated and active TAOK1 and TAOK2 are recognised by the TAOK-pS181 antibody [63] and these phosphorylated proteins are increased in SI fractions prepared from AD brain compared to control brain (Fig. 1b). Additional immunoblotting with phospho-tau and total tau antibodies showed that tau-pS202/S205/S208 (AT8) and tau-pS262/S356 (12E8) were enriched in the sarkosyl-insoluble fraction of AD brain, in which active TAOK-pS181 was also increased (Fig. 1b). Taken together, these results demonstrate that TAOK activity is increased in AD brain compared to controls and this kinase activity co-localises with mild, moderate and severe tau pathology during disease progression. Furthermore, while both TAOK1 and TAOK2 were apparent in the SI fraction of both control and AD brain, active TAOKs appeared to be enriched in SI fraction containing pathological aggregated tau species in AD brain.

### Compound 43 inhibits tau phosphorylation by TAOK2 in vitro and in cells

**Fig. 2:**
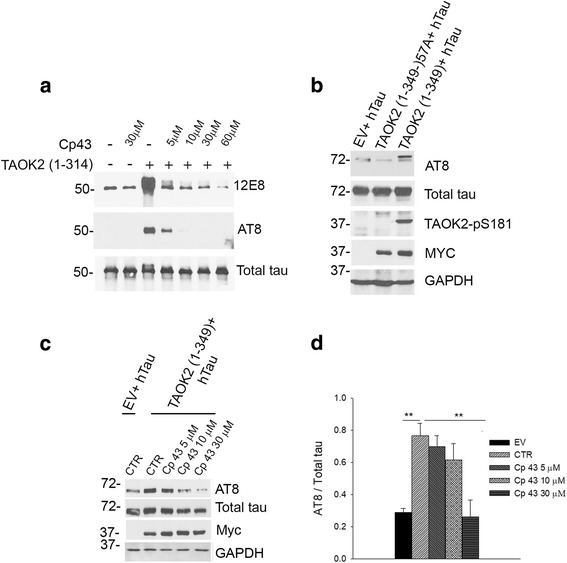
Cp 43 reduced tau phosphorylation by TAOK2. **a** In vitro kinase assays were carried out using GST-TAOK2 (1–314) and/or recombinant htau (2N4R) in the presence or absence of Cp 43 as indicated. Samples were immunoblotted with antibodies to detect tau-pS262/S356 (12E8), tau-pS202/T205/S208 (AT8) or total tau. **b** HEK293T cells were co-transfected with empty vector (EV), kinase-deficient TAOK2 (1–349, K57A) or TAOK2 (1–349) and htau (2N4R) as indicated, and cell lysates immunoblotted with antibodies to detect, TAOK-pS181, tau-pS202/T205/S208 (AT8), total tau, Myc or GAPDH. **c** HEK293T cells were co-transfected with empty vector (EV) or TAOK2 (1–349) and htau (2N4R), and treated with or without Cp 43 as indicated. After 24 h, cell lysates were immunoblotted with antibodies to detect tau-pS202/T205/S208 (AT8), total tau, Myc or GAPDH. **d** Quantitative analysis of the relative levels of tau-pS202/T205/S208 versus total tau in transfected HEK293T cells is shown. The data are normalised to total tau and the bars represent the average ratio ± SEM (*n* = 4); **p < 0.01, one-way ANOVA followed by multiple comparison with the Holm-Sidak method

We have identified a small molecule inhibitor of TAOKs, termed compound 43 (Cp 43) [29]. Cp 43 has an IC_50_ of 11–15 nM, is ATP-competitive and targets TAOKs selectively [29]. In vitro kinase assays were used to determine whether Cp 43 could inhibit the phosphorylation of tau by TAOK2. Purified recombinant TAOK2 (amino acids 1–314) containing the catalytic domain, was incubated with recombinant human tau (htau, 2N4R isoform) in vitro in the presence of increasing concentrations of Cp 43 (5–60 μM) and samples were analysed by immunoblotting with 12E8 and AT8 phospho-tau antibodies. Cp 43 inhibited phosphorylation of tau by TAOK2 in a dose-dependent manner (Fig. 2a).

To investigate the effects of TAOK2 on tau phosphorylation in cells, HEK293T cells were transfected with constructs to express Myc-TAOK2 (1–349) or kinase-defective Myc-TAOK2 (1–349, K57A) and htau (2N4R), and cell lysates were probed with AT8 antibody. Figure 2b shows that exogenous TAOK2, but not kinase-defective TAOK2 (K57A), enhanced tau phosphorylation on the AT8 epitope. HEK293T cells expressing TAOK2 and htau were also incubated with increasing concentrations of Cp 43 (5–30 μM) to determine whether this small molecule was able to inhibit tau phosphorylation by TAOK2 in cells. Figure 2c-d shows that Cp 43 (30 μM) significantly reduced TAOK2-mediated tau phosphorylation on the AT8 epitope. Taken together, these results demonstrate that Cp 43 can inhibit tau phosphorylation by TAOK2 in vitro and in cells.

### TAOKs are expressed and active in differentiating cortical neurons

**Fig. 3:**
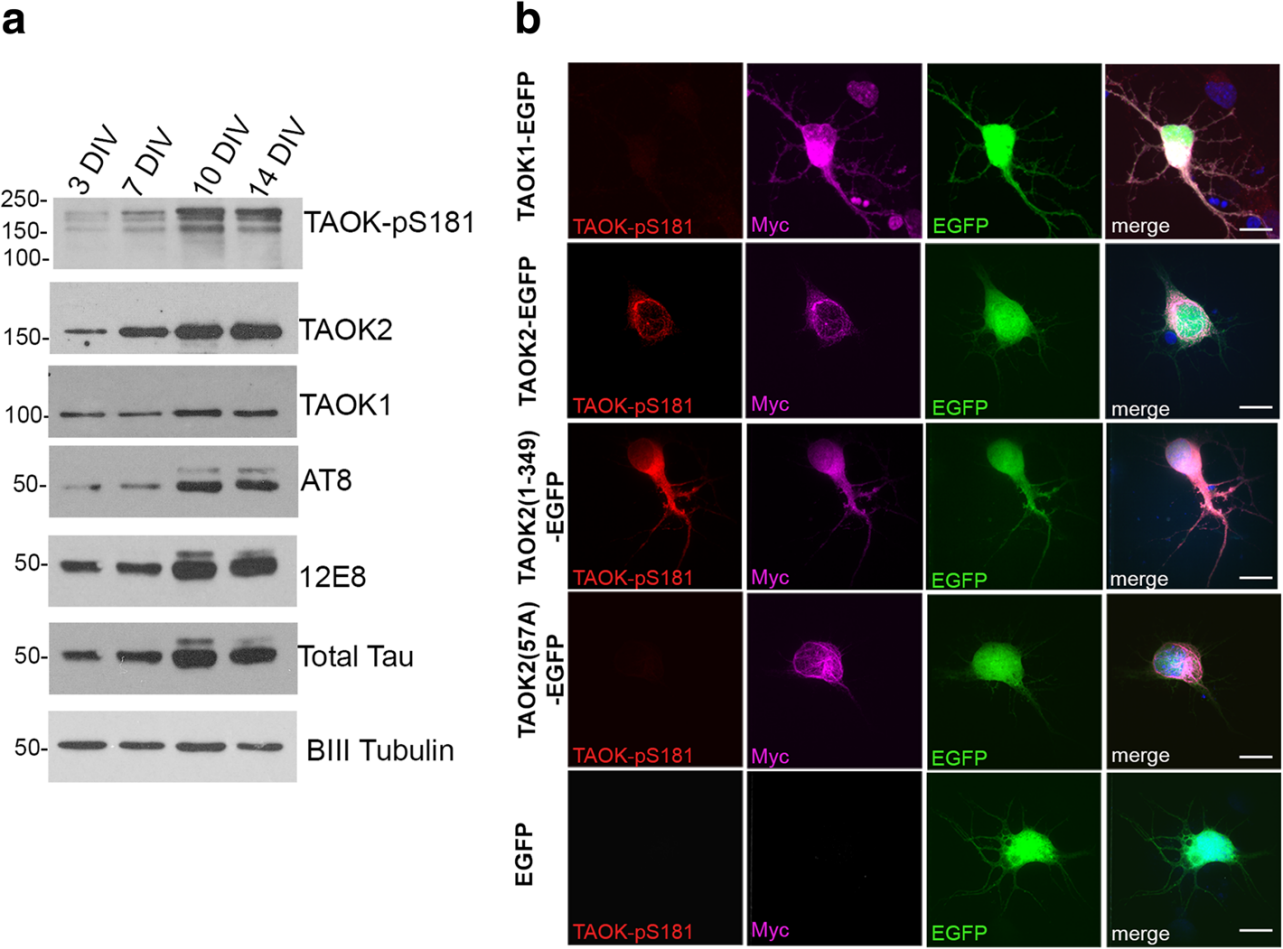
Primary cortical neurons expressed active TAOK-pS181. **a** Neurons were cultured for 3, 7, 10 or 14 DIV and lysates immunoblotted with antibodies to detect TAOK-pS181, TAOK1, TAOK2, tau-pS202/T205/S208 (AT8), tau-pS262/S356 (12E8), total tau or BIII tubulin as indicated. **b** Neurons were transfected with pCAGS-Myc-TAOK1-IRES-EGFP (TAOK1-GFP), pCAGS-Myc-TAOK2-IRES-EGFP (TAOK2-EGFP), pCAGS-Myc-TAOK2–1-349-IRES-EGFP (TAOK2–1-349-EGFP), pCAGS-Myc-TAOK2-K57A-IRES-EGFP (TAOK2 K57A-EGFP) or pCAGS-IRES-EGFP (EGFP) plasmids, as indicated. 7 DIV transfected neurons were fixed and immunostained with antibodies to detect active TAOK-pS181 (red) and Myc (magenta). Nuclei were counterstained with DAPI (blue) and representative maximal projection of confocal z-stack images is shown. Scale bar = 10 μm

Previous studies have demonstrated that TAOK2 is expressed in the brains of developing mouse embryos [10, 59]. Therefore, we investigated TAOK expression and activity during differentiation of rat primary cortical neurons. Lysates prepared from cortical neurons at 3, 7, 10, and 14 days in vitro (DIV) were immunoblotted with antibodies to detect TAOK-pS181, TAOK2, or TAOK1. The activity and expression of TAOKs increased between 3 and 14 DIV relative to βIII-tubulin, in parallel with increases in total and phosphorylated tau recognised by AT8 and 12E8 (Fig. 3a).

To further investigate the activity of TAOKs during differentiation, cortical neurons at 5 DIV were transfected with a bicistronic vector expressing EGFP as a reporter, together with Myc-TAOK1, Myc-TAOK2, the catalytic domain of TAOK2 (amino acids 1–349) or full-length kinase-defective TAOK2 (K57A). Neurons were fixed 48 h after transfection and immunostained with antibodies to detect either Myc (TAOKs) or active TAOK-pS181. Exogenous expression of both TAOK1 and TAOK2 was confirmed by detection of the Myc tag in transfected neurons, however, only TAOK2 was active, as detected by the TAOK-pS181 antibody (Fig. 3b). Full-length TAOK2 (both wild-type and K57A) localised to the perinuclear microtubule cage in differentiating neurons (Fig. 3b), as described previously [43]. In contrast, the catalytic domain of TAOK2 (1–349), which lacks the microtubule binding region (amino acids 745–1235) [43], was distributed throughout the cytoplasm and neurites (Fig. 3b). Expression of full-length and kinase-defective TAOK2 (K57A) in cortical neurons provided a negative control, which was detected by the Myc antibody but not the TAOK-pS181 antibody (Fig. 3b). Exogenous TAOK2 was therefore expressed and catalytically active in transfected cortical neurons.

### TAOK inhibition reduces tau phosphorylation in rat primary cortical neurons

**Fig. 4:**
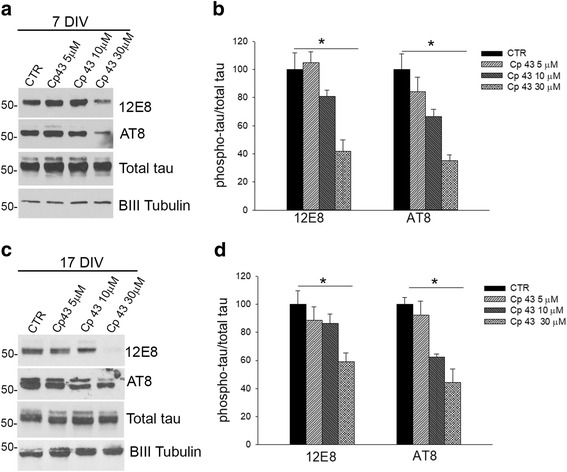
Cp 43 reduced tau phosphorylation in primary cortical neurons. **a**, **c** Lysates were prepared from 7 DIV (**a**) or 17 DIV(**c**) neurons treated with or without Cp 43 for 72 h as indicated and immunoblotted with antibodies to detect tau-pS262/S356 (12E8), tau-pS202/T205/S208 (AT8), total tau or BIII Tubulin. **b**, **d** Quantitative analysis of the effects of increasing Cp 43 concentration on the levels of phosphorylated tau, 12E8 and AT8 epitopes, in 7 DIV (**b**) and 17 DIV neurons (**d**). The data are normalised to total tau and the bars represent the average ratio ± SEM (*n* = 7). *p < 0.05, one-way ANOVA followed by multiple comparison with the Holm-Sidak method

To evaluate the effects of TAOK inhibition on tau phosphorylation in primary rat cortical neurons, cultures at 7 and 17 DIV were treated with Cp 43 (5, 10, 30 μM) for 72 h prior to analysis on western blots. Cp 43 (30 μM) caused significant reductions in tau phosphorylation at the 12E8 and AT8 epitopes, relative to total tau, in neurons cultured for both 7 and 17 DIV (Fig. 4a-d). TAOK inhibition did not increase the number of active caspase-3 positive 7 or 17 DIV neurons treated with Cp 43 (30 μM) for 72 h or affect the expression of the pre-synaptic proteins synapsin 1 and synaptophysin in 17 DIV neurons (Additional file 2: Figure S1 A-C). Cp 43 was able to reduce tau phosphorylation significantly after 6 h of incubation even at a lower concentration of 10 μM (Additional file 2: Figure S1 D-E). The release of lactate dehydrogenase (LDH) into the culture medium was also used to assess cytotoxicity. 7 and 17 DIV neurons were incubated with increasing concentrations of Cp 43 (6 h) and the release of LDH into the culture medium determined. Cp 43 did not affect LDH levels in the media or appear to be cytotoxic under these conditions (Additional file 2: Figure S1F). Taken together, these results show that TAOK inhibition can reduce tau phosphorylation in differentiating neurons without inducing significant cytotoxicity.

### Two new tau residues (T123 and T427) are phosphorylated by TAOK2 and associate with tangles in AD and FTLD-tau brain

Mass spectrometric analysis has identified 49 tau residues that are phosphorylated by TAOK1 and TAOK2 in vitro, including several sites that are phosphorylated aberrantly in AD brain [55]. The phosphorylation of two of these AD-tau residues, T123 and T427, has not yet been attributed to the activity of any alternative kinases and may therefore be specific to TAOKs (https://bit.ly/1SpzgoL). Therefore, we generated two new phospho-tau antibodies to detect tau-pT123 and tau-pT427, and these were used to monitor TAOK activity in human brain and cell experimental models.

**Fig. 5:**
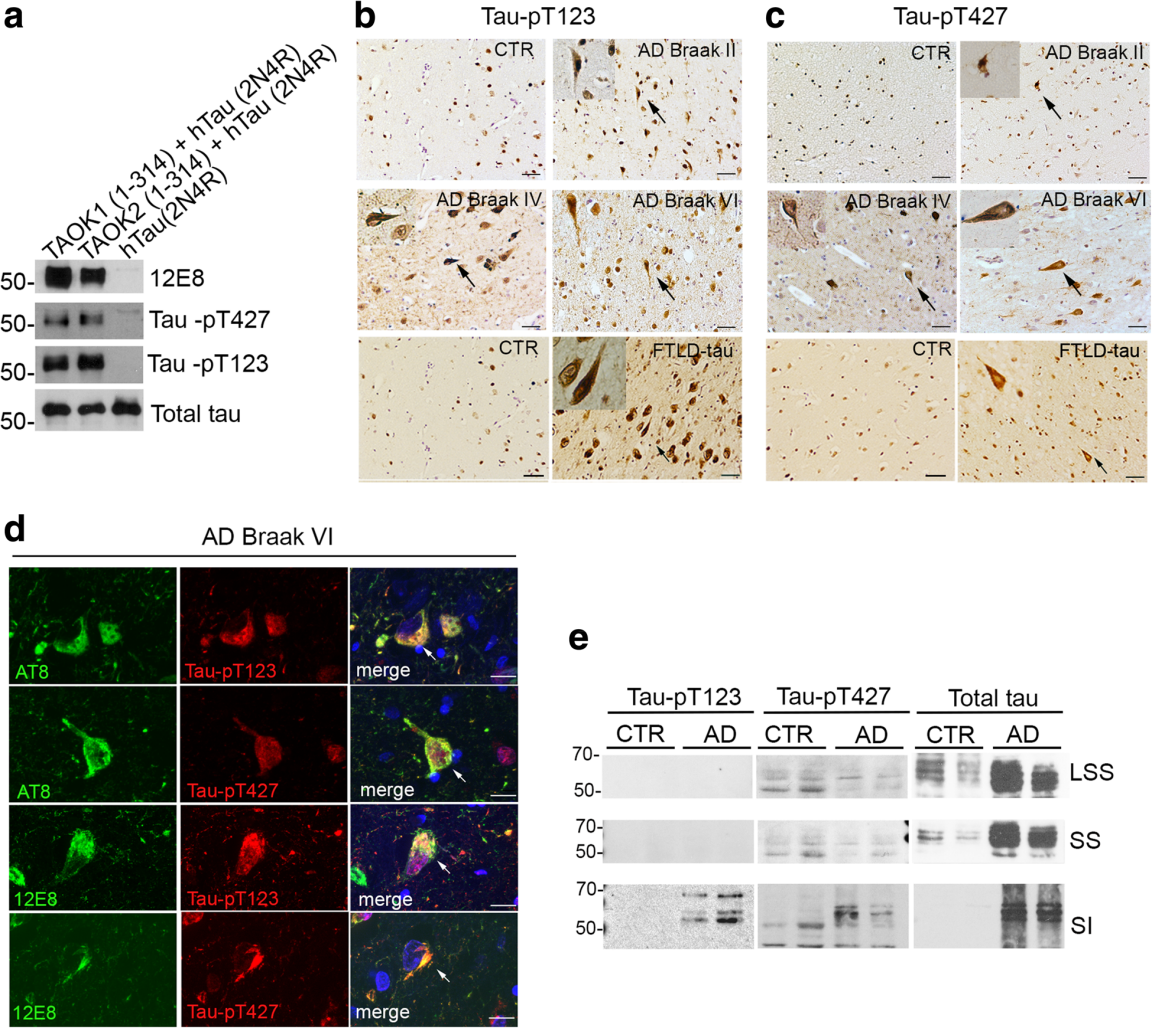
Tau-pT123 and tau-pT427 are targeted by TAOKs and are associated with tangles in AD and FTLD-tau brain. **a** In vitro kinase assays were carried out using GST-TAOK1 (1–314), GST-TAOK2 (1–314) and/or htau (2N4R) as indicated and samples immunoblotted with antibodies to detect tau-pS262/S356 (12E8), tau-pT427, tau-pT123 or total tau. **b-c** Entorhinal cortices from control (CTR), AD (Braak stage II-VI) or FTLD-tau brains were immunostained with antibodies to detect tau-pT123 (**b**) or tau-pT427 (**c**). Strong positive antibody immunoreactivity was observed for tau-pT123 and tau-pT427 at NFTs in each stage of AD and in FTLD-tau (arrows indicate magnified NFTs). **d** Sections from the entorhinal cortex of advanced AD (Braak stage VI) brains were immunostained with antibodies to detect tau-pS202/T205/S208 (AT8, green) or tau-pS262/S356 (12E8, green) and tau-pT123 or tau-pT427 (red) as indicated. Nuclei were counterstained with DAPI (blue). **e** AD (Braak VI) or control (CTR) hippocampal and entorhinal cortex brain samples were extracted with sarkosyl and low speed supernatants (LSS) or sarkosyl-soluble (SS) or sarkosyl-insoluble fractions (SI, insoluble tau) immunoblotted with antibodies to detect tau-pT123, tau-pT427 or total tau as indicated. Representative images and immunoblots are shown. Scale bar = 20 μm

In vitro kinase assays were carried out using recombinant htau (2N4R) and purified TAOK1 (1–314) or TAOK2 (1–314), and phosphorylation of tau on both sites was detected on western blots using antibodies tau-pT123, tau-pT427 and 12E8 (Fig. 5a). Entorhinal cortex sections (× 12 per brain sample) from brains presenting mild (Braak stage II, *n* = 3), moderate (Braak stage IV, *n* = 3) or severe (Braak stage VI, *n* = 4) AD pathology, or from FTLD-tau brains (*n* = 2) or control brains (*n* = 4), were immunostained with the tau-pT123 and tau-pT427 antibodies. Tangle-like structures were labelled with both the tau-pT123 and tau-pT427 antibodies in all AD (Braak stages II, IV and VI) and FTLD-tau sections (Fig. 5b-c). Immunoreactivity was also detected in some nuclei in AD and FTLD-tau brain and to a lesser extent in controls (Fig. 5b-c).

Epitope specificity for the tau-pT123, tau-pT427 and TAOK-pS181 antibodies was confirmed by pre-treating these reagents with their appropriate phospho-peptides, which blocked antibody recognition of their target epitopes in immunostained AD sections and in immunoblotted in vitro kinase assays (Additional file 3: Figure S2A-B). Tau-pT123 and tau-pT427 antibody labelling co-localised with 12E8 and AT8 in AD sections, confirming labelling of tangles by both of the new phospho-dependent tau antibodies (Fig. 5d). Extracts from the entorhinal cortex of AD (Braak VI; *n* = 2) and control brains (*n* = 2) showed that tau-pT123 and tau-pT427 were enriched in the sarkosyl insoluble (SI) fractions extracted from AD but not control brains (Fig. 5e). The results presented here confirm that both of these tau residues are targeted by TAOKs and show that tau is phosphorylated on T123 and T427 in early and late-stage AD and also in FTLD-tau brain.

### TAOK2 activity is required to phosphorylate tau on T427 in cortical neurons

**Fig. 6:**
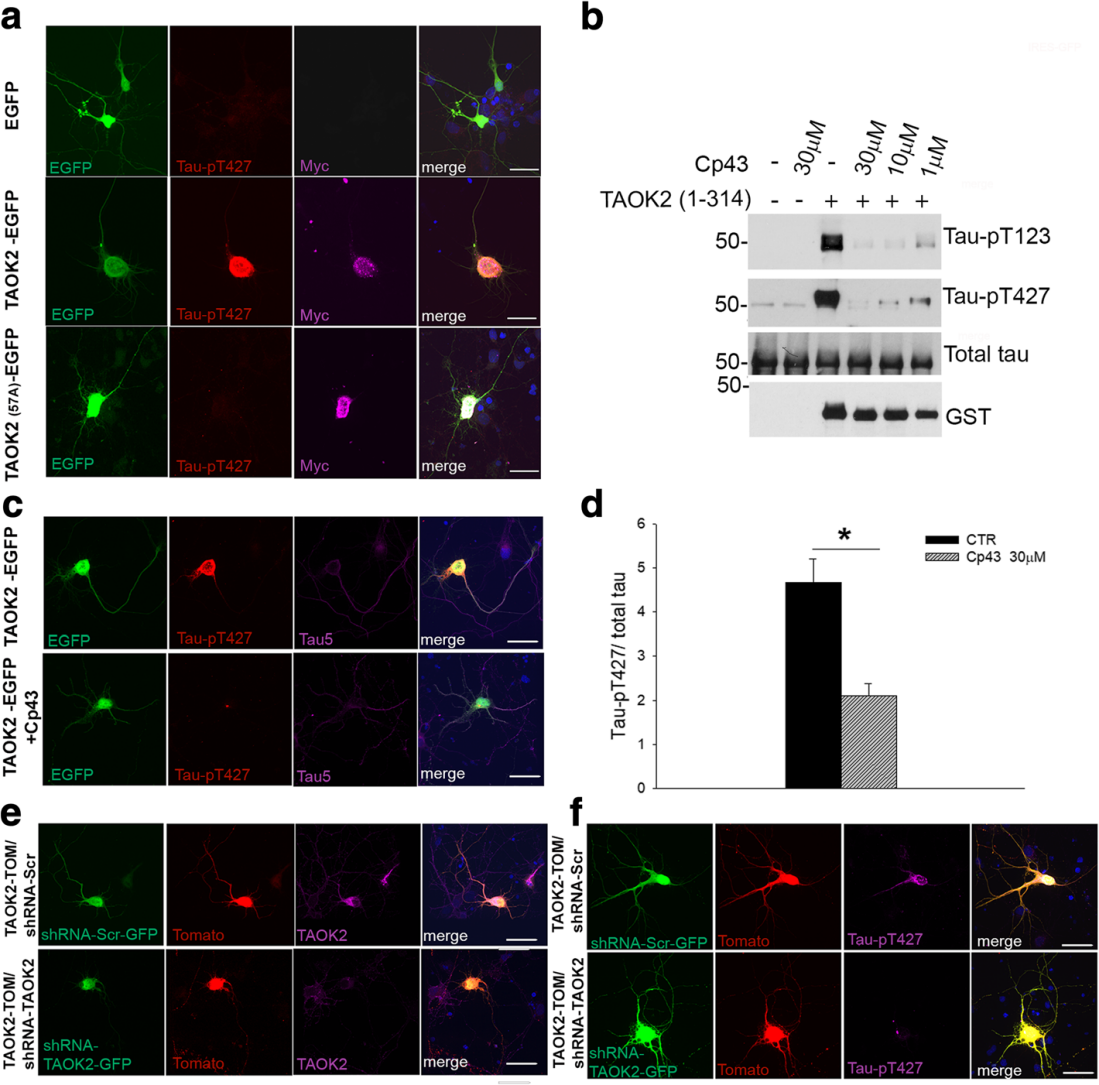
Cp 43 reduced tau phosphorylation on T427 by TAOK2 in primary neurons. **a** IRES-EGFP and TAOK2-EGFP transfected neurons (7 DIV) were fixed and immunostained with antibodies to detect tau-pT427 (red) and Myc (magenta). **b** In vitro kinase assays were carried out using GST-TAOK2 (1–314) and/or recombinant htau (2N4R) in the presence or absence of Cp 43 as indicated. Samples were immunoblotted with antibodies to detect tau-pT123, tau-pT427, total tau or GST. **c** TAOK2-EGFP transfected neurons (7 DIV) were incubated with or without Cp 43 (30 μM) for 24 h as indicated and immunostained with antibodies to detect tau-pT427 (red) or total tau (tau-5, magenta). **d** Quantitative analysis of tau-pT427 immunoreactivity in TAOK2-EGFP transfected neurons incubated with or without Cp 43. Bars represent the average immunofluorescence intensity ± SEM of tau-pT427 normalised to total tau. > 65 neurons were analysed for each group (*n* = 4). *p < 0.05, student t-test. **e**-**f** TAOK2-IRES-Tomato and TAOK2-shRNA-EGFP or scrambled-shRNA-EGFP were co-transfected into neurons as indicated and after 72 h cells were fixed and immunostained for TAOK2 (magenta, **e**) or tau-pT427 (magenta, **f**). Nuclei were counterstained with DAPI (blue). Representative maximal projection of z-stack images and immunoblots are shown. Scale bar = 10 μm

Tau phosphorylation on T123 and T427 was investigated next in rat primary cortical neurons using a bi-cistronic IRES vector to express Myc-TAOK2 together with EGFP to provide a reporter protein. 7 DIV transfected neurons expressing exogenous active TAOK2 were strongly immunoreactive with the tau-pT427 antibody (Fig. 6a). In contrast, untransfected neurons or transfected neurons expressing kinase-defective TAOK2 (K57A) or EGFP alone were not recognised by the tau-pT427 antibody (Fig. 6a). These results demonstrate that tau is not phosphorylated physiologically on T427 in differentiating cortical neurons. The ability of Cp 43 to inhibit tau phosphorylation on T123 and T427 by TAOK2 was initially tested using in vitro kinase assays, which showed that tau phosphorylation on these epitopes was decreased by the inhibitor compound (Fig. 6b). Treatment of TAOK2 transfected cortical neurons with Cp 43 (30 μM, 24 h) also reduced tau phosphorylation on T427, and tau-pT427 immunoreactivity was decreased by 55% in neurons incubated with the inhibitor compared to controls (Fig. 6c-d, Cp 43 *n* = 70, controls *n* = 65). In addition, co-transfection of Myc-TAOK2-IRES-Tomato together with TAOK2-shRNAs to knockdown expression of exogenous TAOK2 in cortical neurons (Fig. 6e) also decreased tau-pT427 immunoreactivity (Fig. 6f). These results show that tau phosphorylation on T427 was stimulated by expression of exogenous TAOK2 in transfected neurons and that this effect can be inhibited by treating cells with Cp 43 or by silencing TAOK2 expression using shRNAs.

Although we did not observe any positive immunoreactivity in lysates from rat primary cortical neurons immunoblotted with tau-pT123 and tau-pT427 antibodies, tau-pT123 immunostaining showed a positive signal in untransfected neurons that was restricted to the nuclear compartment. However, this signal was not increased following transfection and over-expression of TAOK2 (Additional file 4: Figure S3A).

### Cp 43 decreased tau phosphorylation in murine and human neuronal cell models of tauopathy

**Fig. 7:**
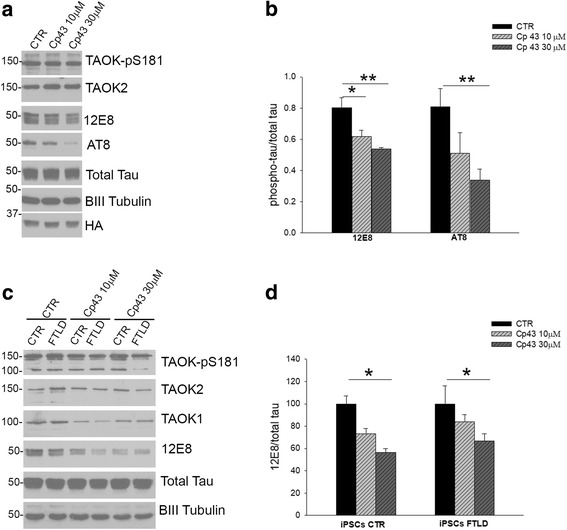
Cp 43 decreased tau phosphorylation in murine and human neuronal models of tauopathy. **a** Neurons were prepared from Tau35 transgenic mouse embryos and incubated at 14 DIV with or without Cp 43 (10, 30 μM, 6 h) and lysates immunoblotted with antibodies to detect TAOK-pS181, TAOK2, tau-pS262/S356 (12E8), tau-pS202/T205/S208 (AT8), total tau, BIII Tubulin or HA. **b** Quantitative analysis of the relative levels of phosphorylated tau (12E8 and AT8) in Tau-35 primary neurons. The data are normalised to total tau and the bars represent the average ratio ± SEM (*n* = 6). **c** CTR or FTLD (10 + 16 *MAPT* mutation) iPSC derived neurons (60 DIV) were incubated with or without Cp 43 (10, 30 μM, 6 h) and lysates immunoblotted with antibodies to detect TAOK-pS181, TAOK1, TAOK2, tau-pS262/S356 (12E8), total tau or BIII Tubulin. **d** Quantitative analysis of the relative levels of phosphorylated tau (12E8) present in Cp 43 treated iPSC derived neurons. Data are normalised to total tau and are expressed as mean percentages ± SEM of the controls (*n* = 6). *p < 0.05; **p < 0.01, one-way ANOVA followed by multiple comparison using the Holm-Sidak method

The positive immunoreactivity for TAOK-pS181, tau-pT123 and tau-pT427 antibodies observed in the tangle structures in FTLD-tau-brain suggests that TAOK activity may contribute to tau pathology in this disease. Therefore, we decided to evaluate the effect of TAOK inhibition on tau phosphorylation in murine and human neuronal cell models of FTLD-tau. Previous studies have demonstrated the presence of an N-terminally truncated form of tau in tauopathy brains [64]. This human brain derived tau fragment, tagged with HA to provide an epitope tag, has recently been used to generate the Tau-35 mouse model, which displays key disease associated features including enhanced tau phosphorylation and the presence of tangle like structures in the brain [4, 54]. The activity of TAOKs and their potential contribution to tau phosphorylation was investigated here by preparing primary cortical neurons from Tau35 transgenic mouse embryos and incubating these cultures in the presence or absence of Cp 43 (10 or 30 μM for 6 h). Cell lysates were prepared at 14 DIV and western blotting analysis detected active TAOK-pS181 and demonstrated that treatment with Cp 43 caused significant reductions in tau phosphorylation on the 12E8 and AT8 epitopes (Fig. 7a-b). Tau did not appear to be phosphorylated on T123 or T427 in these lysates prepared from Tau35 mouse neurons and this result was similar to our observations in wild-type rat primary cortical neurons.

To investigate the effects of TAOKs on tau phosphorylation in human neurons, induced pluripotent stem cells (iPSCs) from two control iPSC lines and one iPSC line generated from an individual with the FTLD-associated 10 + 16 *MAPT* mutation, were differentiated into cortical neurons [53]. Cells were cultured for 60 DIV and neuronal morphology was analysed by expression of MAP2, total tau, unphosphorylated tau (Tau1) and phosphorylated tau (12E8) (Additional file 5: Figure S4). Lysates prepared from these cells showed similar levels of expression of TAOK1, TAOK2 and active TAOK-pS181 were detected in control and FTLD-tau samples (Fig. 7c). Tau phosphorylation on the 12E8 epitope was comparable in control and FTLD-tau neurons at 60 DIV (Fig. 7c). Treatment with Cp 43 (30 μM, 6 h) caused a significant reduction in 12E8 immunoreactivity in both control and FTLD-tau human neurons (Fig. 7c-d). These results show that endogenous TAOKs are active in these murine and human neuronal models for tauopathy and that tau phosphorylation was decreased by Cp 43.

## Discussion

An important objective for dementia research is the identification of tau kinases that are catalytically active at tangle structures in tauopathies and therefore likely to contribute directly to the aberrant phosphorylation and aggregation of tau. In this study, active TAOK-pS181 was present and detected at pre-tangles and tangles in AD brain sections displaying mild, intermediate and advanced tau pathology (Braak stages II-VI). In contrast, we were unable to detect significant TAOK activity in control brain sections. Sarkosyl-insoluble extracts of AD brain tissues (Braak stage VI) also demonstrated the presence of active TAOK-pS181 in those fractions which contained aggregated and pathologically phosphorylated tau, whereas TAOK activity and phosphorylated tau were largely absent in extracts prepared from control brain.

These results are consistent with potential roles for TAOKs in the development of early and late-stage tau pathology in AD brain. The association between aberrant phosphorylation and self-aggregation of tau in pathological conditions has been well documented, however, the precise phosphorylation events resulting in tau toxicity are not well defined [14, 37]. KXGS motifs located within the tau repeat domain are of special interest because tau phosphorylation on the 12E8 epitope (S262/S356) reduces tau-microtubule binding and contributes to the pathogenic cascade, where tau is phosphorylated on additional residues associated with tangle formation [22, 45]. A recent study has also shown that tau phosphorylation on the AT8 epitope (S202/T205/S208), located within the proline rich domain, is sufficient to promote tau aggregation and relocalisation of pathogenic tau in the dendrite compartment, and these events occur during early neurodegeneration in AD [11, 27]. Attenuation of tau phosphorylation on these sites, through inhibition of appropriate tau kinases, could provide a suitable strategy for the treatment of early stage AD and related tauopathies.

Abnormal tau phosphorylation can result in aberrant pathology via several different mechanisms, and these appear to include changes in tau degradation pathways and cleavage. For example, tau phosphorylated on S262/S356 is not recognised by the C terminus of HSP70-interacting protein-heat shock protein 90 (CHIP–HSP90) complex and is therefore protected from proteasomal degradation [12]. In addition, tau phosphorylation on S422 can prevent tau cleavage by Caspase 3 at T421 [18]. We have demonstrated previously that TAOKs can phosphorylate tau on more than 40 residues in vitro*,* and 29 of these sites are modified in PHF-tau extracted from AD brain [55]. Two of these PHF-tau residues, T123 and T427, were investigated here because they are uncharacterised and could be targeted by TAOKs specifically. Immunohistochemical analysis of early and late stage AD brain sections (Braak II-VI) detected tau-pT123 and tau-pT427 at pre-tangles and tangles, where TAOK activity also occurred. Furthermore, sarkosyl-insoluble tau from AD brain was also phosphorylated on T123 and T427 and this fraction also contained active TAOK-pS181.

The presence of TAOK activity at pre-tangles and tangles in early stage AD brain correlates with the appearance of tau phosphorylated on multiple epitopes recognised by the 12E8, AT8, tau-pT123 and tau-pT427 antibodies. The ability of TAOKs to phosphorylate tau on pathogenic epitopes was investigated here using a recently characterised TAOK inhibitor compound, which inhibited tau phosphorylation in vitro and in HEK293 cells expressing exogenous TAOK2 and human tau. Cp 43 also decreased basal levels of tau-12E8 and tau-AT8 in differentiating rat neurons without inducing cytotoxicity or causing significant changes in pre-synaptic protein expression levels in primary neurons.

The normal physiological functions of TAOKs in adult brain remain to be determined but TAOK2 can regulate dendrite arborisation, spine maturation and axonal outgrowth in the developing mouse brain [10, 49, 59, 65]. Here we have shown that endogenous TAOK activity and tau-12E8 and tau-AT8 are increased as primary cortical neurons undergo differentiation and that TAOK2, but not TAOK1, is active catalytically when over-expressed in transfected neurons. Furthermore, TAOK-pS181 immunoreactivity in lysates prepared from rat cortical neurons was observed and this antibody recognised a protein band that appeared to migrate with the same mobility as TAOK2. These observations are consistent with a role for TAOK2 in regulating neuronal maturation during early murine brain development as reported previously [10, 49, 59, 65]. TAOK expression and activity were also detected in differentiating Tau35 primary neurons and in FTLD-tau and control human iPSC-derived neurons. However, only limited immunoreactivity with the TAOK-pS181 antibody was observed in adult human asymptomatic brain sections when compared to AD and FTLD brains, where strong TAOK-pS181 immunoreactivity occurred at tangle structures. These results suggest that appropriate levels of TAOK activity are required for neuronal maturation in the developing brain whereas increased TAOK activity in the adult brain may result in pathogenic effects.

In differentiating primary cortical neurons tau was not phosphorylated on T427 unless these cells were transfected with TAOK2. Tau was however phosphorylated on T123 where immunoreactivity was located in the nucleus of differentiating neurons and phosphorylation levels on this epitope were not increased by over-expression of TAOK2. Previous studies have demonstrated enriched expression of the 1N4R tau isoform in the neuronal nucleus but not in the cytoplasm or neurites, where the 0N3R fetal tau isoform is predominantly expressed at this stage of differentiation [6, 35, 42]. The absence of mature tau isoform expression in the cytoplasm and neurites in differentiating neurons and the lack of TAOK2 expression in the nucleus, may account for the inefficient phosphorylation of tau on T123 by TAOK2 observed here.

Tau phosphorylation and TAOK activity were also examined in an additional tauopathy. Immunohistochemical analysis of FTLD-tau brain sections showed that active TAOK-pS181, tau-pT123 and tau-pT427 each localised to pre-NFTs and NFT structures. Primary cortical neurons were also prepared from brains from Tau35 transgenic mice, which overexpress a C-terminally truncated 35 kDa htau fragment that is associated with tauopathies [4]. Treatment of these Tau35 mouse neurons with Cp 43 reduced tau phosphorylation on the 12E8 and AT8 epitopes. Human neurons were also produced using iPSCs derived from control and individual with the 10 + 16 *MAPT* mutation which is associated with FTLD and these differentiated cells expressed active TAOKs and tau phosphorylated on S262/S356. Addition of Cp 43 decreased tau phosphorylation on the 12E8 epitope in these FTLD iPSC derived neurons but we were unable to detect tau-pT123 or tau-pT427. A previous study has shown that these FTLD neurons express the foetal 0N3R tau isoform at this time point (60 DIV) and it is possible that adult tau isoform expression and enhanced TAOK activity are required for tau phosphorylation on T123 and T427 [53]. In contrast, T123 and T427 were phosphorylated under pathogenic conditions in FTLD-tau brain sections, where all six tau isoforms are expressed, and tau-pT123 and tau-pT427 localised to pre-tangles and tangles, where TAOK activity occurred.

A number of kinases can phosphorylate tau in vitro and to date GSK-3, MARK, JNK, p38, AMPK*,* Nuak1 and TAOKs have been shown to be active catalytically at tangles in AD brain tissues [15, 21, 31, 57, 60, 67]. TAOKs can stimulate the catalytic activity of several of these tangle-associated kinases, including MARK, JNK and p38, and TAOKs could potentially phosphorylate tau on additional pathological sites via such pathways [25, 44, 56]. Indeed, TAOKs can phosphorylate and activate MARK directly and both of these kinases can phosphorylate tau on the 12E8 epitope [3, 56]. The appearance of TAOK activity at pre-tangle and tangle structures during early AD (Braak stage II) and their phosphorylation of tau on multiple residues, including pathogenic sites such as T123 and T427, are consistent with potential roles for TAOKs during early disease development. Therefore, inhibitors of TAOK activity may reduce aberrant tau phosphorylation and tau pathology. In a previous study using breast cancer cell models, we have shown that abnormal TAOK activity can contribute to clustering of supernumerary-amplified centrosomes in malignant cells and prevents multipolar mitoses and associated cell death [29]. Cp 43 can stimulate centrosome declustering, multipolarity and mitotic catastrophe in these centrosome amplified cancer cells selectively, whereas normal bipolar cells continue to divide and proliferate in the presence of this compound [29]. Aberrant TAOK activity can therefore induce clustering of supernumerary-amplified centrosomes in malignant breast cells as well as stimulating pathological tau phosphorylation in neurons and tauopathies and TAOKs may therefore offer suitable therapeutic targets for both diseases. To date, TAOKs are not reported to be involved in any other diseases.

Tau kinase inhibitors have been used already to treat tau transgenic mice, in order to slow or prevent the development of tau pathology in these animals [28, 32, 46]. Previous studies have shown that inhibiting GSK3 reduces tau phosphorylation and ameliorates memory deficits in transgenic mouse models of AD [7, 46, 47, 51]. Initial studies targeting GSK-3 produced encouraging results in pre-clinical studies but failed during clinical trials due to off-target toxicity and the requirement for GSK-3 activity to sustain normal cell function [39]. The appearance of TAOK activity at NFTs in AD and FTLD, and their phosphorylation of tau on multiple pathological sites, suggests that these proteins could offer novel and suitable alternative drug targets for the treatment of neurodegenerative disorders.

## Conclusions

In summary, we have shown that TAOKs are active and co-localise with tangle structures in AD and FTLD brain. We have used a new TAOK inhibitor to reduce tau phosphorylation on sites associated with neurodegeneration in human tauopathies and examined two new phosphorylation sites on tau (T123 and T427) that are targeted by TAOKs and detected in AD and FTLD brain. TAOK activity and tau phosphorylation on pathological sites are also shown to be decreased by Cp 43 in vitro and in neuronal and disease cell models. Our results suggest compounds that inhibit the activity of these kinases could reduce tau pathology and potentially delay associated cognitive decline with such effects providing significant benefits for individuals with early symptoms of dementia.

## Additional files

Additional file 1: **Table S1.** Characteristics of the subjects whose brain tissues were used in this study. PMD refers to post-mortem delay in hours before samples were processed. (DOCX 16 kb)

Additional file 2: **Figure S1.** Cp 43 decreased tau phosphorylation in rat primary cortical neurons. **A.** Quantitative analysis of cleaved active Caspase 3 positive neurons (7 DIV or 17 DIV) treated with or without 30 μM Cp 43 (72 h). > 800 neurons were counted for each experimental condition and bars represent mean percentages ± SEM (*n* = 4). **B.** Neurons (14 DIV) were incubated without or with Cp 43 (5, 10 or 30 μM, 72 h) and lysates immunoblotted with antibodies to detect Synapsin 1, Synaptophysin or BIII tubulin. **C.** Quantitative analysis of the effects of Cp 43 on synaptic protein expression levels normalised to BIII tubulin. Bars represent the average ratio ± SEM (*n* = 7). **D.** Neurons (7 DIV) were treated with or without Cp 43 for 6 h as indicated and lysates immunoblotted with antibodies to detect tau-pS262/S356 (12E8), tau-pS202/T205/S208 (AT8), total tau or BIII Tubulin. **E.** Quantitative analysis of the effects of increasing Cp 43 concentration on the levels of phosphorylated tau (12E8 and AT8 epitopes). Data are normalised to total tau and are expressed as mean percentages ± SEM of the controls (*n* = 5). ** *p* < 0.01 *p < 0.05, one-way ANOVA followed by multiple comparison with the Holm-Sidak method. **F.** 7 and 17 DIV neurons were incubated with or without Cp 43 (1–30 μM) for 6 h. Bars represent the average ratio of the percentages of LDH released versus total LDH ± SEM (*n* = 24; collected in 3 independent experiments). Representative immunoblots and images are shown. Scale bar = 10 μm. (TIF 815 kb)

Additional file 3: **Figure S2.** Blocking phospho-peptides abolished epitope recognition by TAOK-pS181, tau-pT123 and tau-p427 antibodies. **A.** Entorhinal cortex sections from AD (Braak VI) brains were immunostained with antibodies to detect TAOK-pS181, tau-pT123 or tau-pT427 in the presence (left side) or absence (right side) of their appropriate blocking phospho-peptide epitopes (1 μM). **B.** In vitro kinase assays were carried out using GST-TAOK1 (1–314) or GST-TAOK2 (1–314) and recombinant htau (2N4R), and samples immunoblotted with tau-pT123 or tau-pT427 antibodies in the presence (right side) or absence (left side) of their appropriate blocking phospho-peptide. Representative images and immunoblots are shown. Scale bar = 20 μm. (TIF 7890 kb)

Additional file 4: **Figure S3.** Tau phosphorylated on T123 was present in the nuclei of rat primary neurons. **A** Neurons (7 DIV) were transfected with IRES-GFP, TAOK2-IRES-GFP or TAOK2 K57A-IRES-GFP, fixed and immunostained with antibodies to detect tau-pT123 (red) and Myc (magenta). Nuclei were counterstained with DAPI (blue). Representative images are shown. Scale bar = 10 μm. (TIF 409 kb)

Additional file 5 **Figure S4** FTLD and control iPSC-derived neurons expressed phosphorylated tau. **A-D.** FTLD (10 + 16 *MAPT* mutation) and control iPSC-derived neurons (60 DIV) were fixed and immmunostained with antibodies to detect total tau (red, A-B), tau-12E8 (green, A-B), tau-1 (green, C-D) and Map2 (red, C-D). Nuclei were counterstained with DAPI (blue). Representative images are shown. Scale bar = 10 μm. (TIF 4365 kb)

## Abbreviations

AD: Alzheimer’s disease
CDK5: cyclin-dependent kinase 5
Cp 43: Compound 43
DAPI: 4′, 6′-diamidino-2-phenylindole
DMEM: Dulbecco’s modified Eagles medium
DMSO: dimethyl sulfoxide
ERK: extracellular-signal regulated kinase
FTLD: Frontotemporal lobar degeneration
GAPDH: glyceraldehyde-3-phosphate dehydrogenase
GFP: green fluorescent protein
GSK-3: Glycogen synthase kinase-3
GST: glutathione-S-transferase
HRP: horse radish peroxidase
iPSC: induced pluripotent stem cell
JNK: c-Jun N-terminal kinase
LDH: lactate dehydrogenase
LSS: low speed supernatant
MAP: microtubule-associated protein
MAPK: mitogen-activated protein kinase
MARK: microtubule-regulated protein kinase
NFT: neurofibrillary tangles
PBS: phosphate buffered saline
PSD95: post synaptic density protein 95
SI: sarkosyl insoluble
SS: sarkosyl soluble
TAOK: thousand-and-one amino acid kinase
