# Supplementary material for: A new TAO kinase inhibitor reduces tau phosphorylation at sites associated with neurodegeneration in human tauopathies
Source: Acta Neuropathol Commun. 2018 May 7;6:37. doi: 10.1186/s40478-018-0539-8 (PMC5937037; doi:10.1186/s40478-018-0539-8)
Supplement: Supplementary file 1 — Table S1. Characteristics of the subjects whose brain tissues were used in this study. PMD refers to post-mortem delay in hours before samples were processed. (DOCX 16 kb) [file 40478_2018_539_MOESM1_ESM.docx]

Table S1: Characteristics of the subjects whose brain tissues were used in this study. PMD refers to post-mortem delay in hours before samples were processed.

|  | **Pathology diagnosis** | **Braak**  **stage** | **Age** | **Sex** | **PMD**  **(h)** |
| --- | --- | --- | --- | --- | --- |
| 1 | AD | VI | 89 | M | 76 |
| 2 | AD | VI | 74 | F | 20 |
| 3 | AD with moderate amyloid angiopathy | VI | 75 | M | 69 |
| 4 | AD with moderate amyloid angiopathy | VI | 75 | M | 69 |
| 5 | AD with moderate amyloid angiopathy and moderate cerebrovascular disease | IV | 79 | M | 29 |
| 6 | AD with mild amyloid angiopathy | IV | 92 | M | 70 |
| 7 | AD with extensive severe amyloid angiopathy | IV | 86 | M | 52 |
| 8 | Mild AD changes | II | 96 | M | 33 |
| 9 | Mild AD changes with extensive amyloid angiopathy | II | 91 | F | 36 |
| 10 | Mild AD changes | II | 91 | F | 36 |
| 11 | FTLD-tau (FTDP-17) |  | 63 | F | 91 |
| 12 | FTLD-tau (strongly suggestive of FTDP-17) with Pick bodies and some focal TDP43 positivity together with extensive p62 positive |  | 70 | F | 16 |
| 13 | Control minimal ageing changes |  | 66 | F | 78 |
|  |  |  |  |  |  |
| 14 | Control minimal ageing changes |  | 73 | F | 27 |
| 15 | Control |  | 43 | F | 43 |
| 16 | Control |  | 77 | M | 11 |
| 17 | Control |  | 51 | F | ND |
| 18 | Control |  | 58 | M | 58 |
