# Supplementary figures and images for: A new TAO kinase inhibitor reduces tau phosphorylation at sites associated with neurodegeneration in human tauopathies
Source: Acta Neuropathol Commun. 2018 May 7;6:37. doi: 10.1186/s40478-018-0539-8 (PMC5937037; doi:10.1186/s40478-018-0539-8)

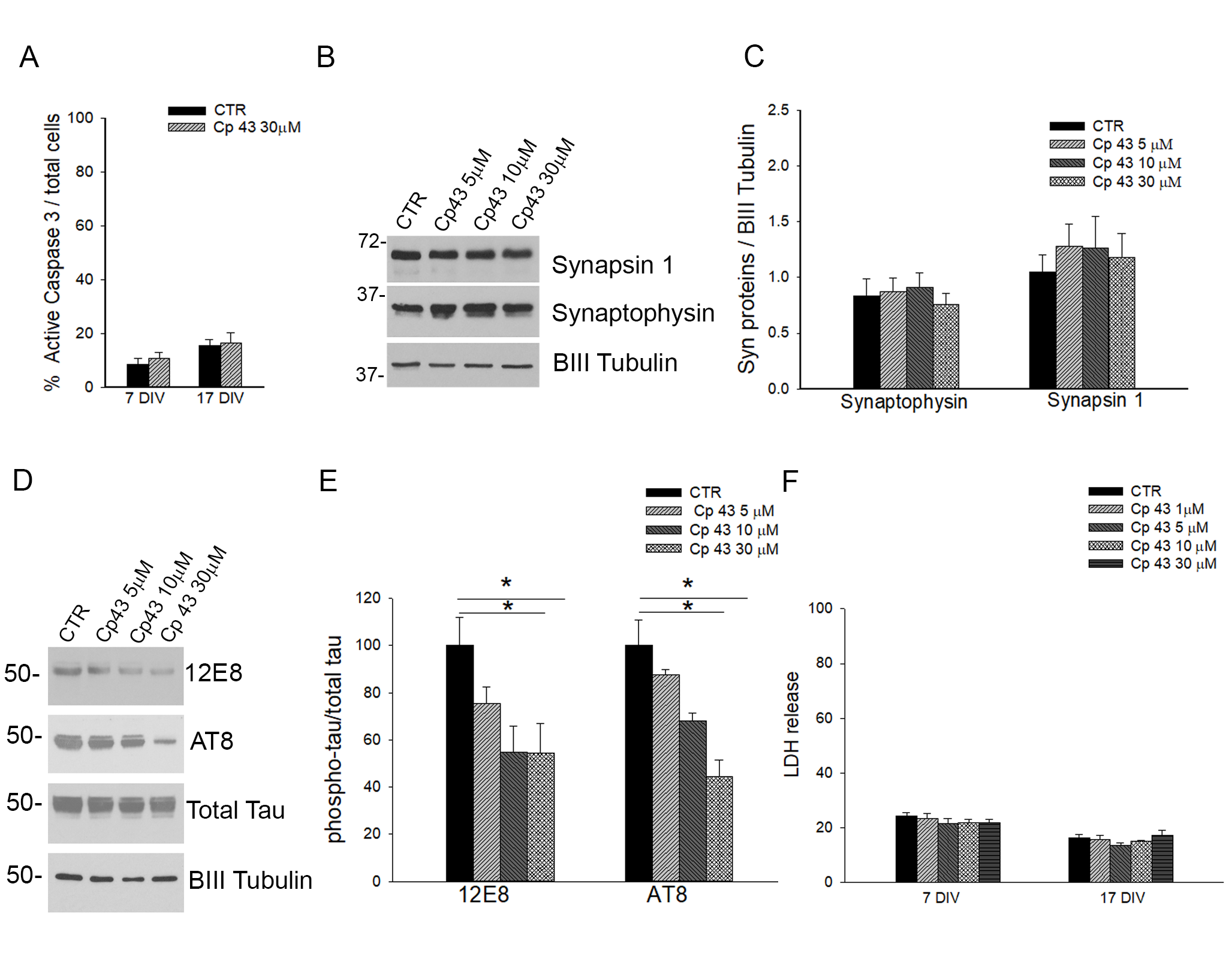

Supplement: Supplementary file 2 — Figure S1. Cp 43 decreased tau phosphorylation in rat primary cortical neurons. A. Quantitative analysis of cleaved active Caspase 3 positive neurons (7 DIV or 17 DIV) treated with or without 30 μM Cp 43 (72 h). > 800 neurons were counted for each experimental condition and bars represent mean percentages ± SEM (n = 4). B. Neurons (14 DIV) were incubated without or with Cp 43 (5, 10 or 30 μM, 72 h) and lysates immunoblotted with antibodies to detect Synapsin 1, Synaptophysin or BIII tubulin. C. Quantitative analysis of the effects of Cp 43 on synaptic protein expression levels normalised to BIII tubulin. Bars represent the average ratio ± SEM (n = 7). D. Neurons (7 DIV) were treated with or without Cp 43 for 6 h as indicated and lysates immunoblotted with antibodies to detect tau-pS262/S356 (12E8), tau-pS202/T205/S208 (AT8), total tau or BIII Tubulin. E. Quantitative analysis of the effects of increasing Cp 43 concentration on the levels of phosphorylated tau (12E8 and AT8 epitopes). Data are normalised to total tau and are expressed as mean percentages ± SEM of the controls (n = 5). ** p < 0.01 *p < 0.05, one-way ANOVA followed by multiple comparison with the Holm-Sidak method. F. 7 and 17 DIV neurons were incubated with or without Cp 43 (1–30 μM) for 6 h. Bars represent the average ratio of the percentages of LDH released versus total LDH ± SEM (n = 24; collected in 3 independent experiments). Representative immunoblots and images are shown. Scale bar = 10 μm. (TIF 815 kb) [file 40478_2018_539_MOESM2_ESM.tif]

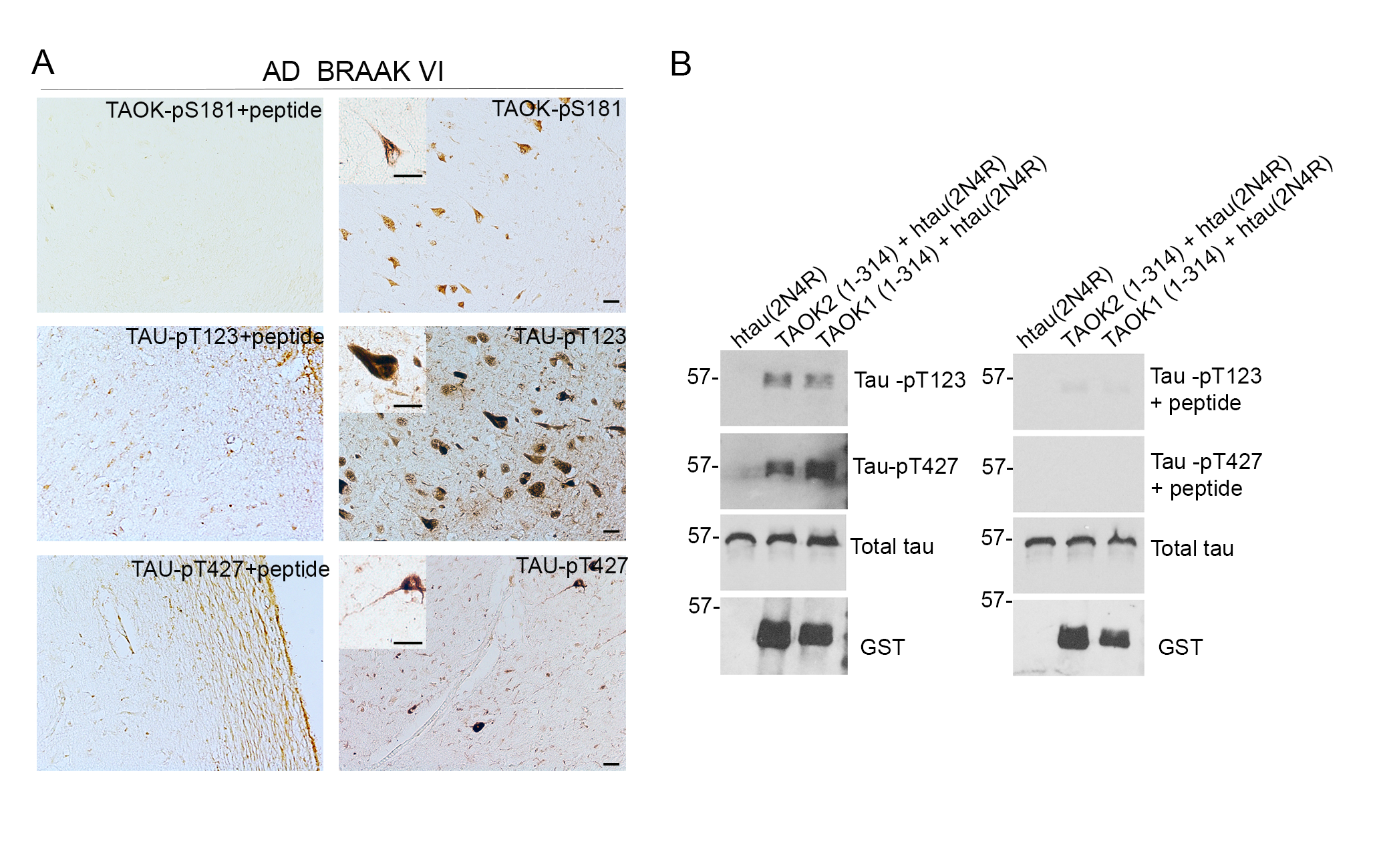

Supplement: Supplementary file 3 — Figure S2. Blocking phospho-peptides abolished epitope recognition by TAOK-pS181, tau-pT123 and tau-p427 antibodies. A. Entorhinal cortex sections from AD (Braak VI) brains were immunostained with antibodies to detect TAOK-pS181, tau-pT123 or tau-pT427 in the presence (left side) or absence (right side) of their appropriate blocking phospho-peptide epitopes (1 μM). B. In vitro kinase assays were carried out using GST-TAOK1 (1–314) or GST-TAOK2 (1–314) and recombinant htau (2N4R), and samples immunoblotted with tau-pT123 or tau-pT427 antibodies in the presence (right side) or absence (left side) of their appropriate blocking phospho-peptide. Representative images and immunoblots are shown. Scale bar = 20 μm. (TIF 7890 kb) [file 40478_2018_539_MOESM3_ESM.tif]

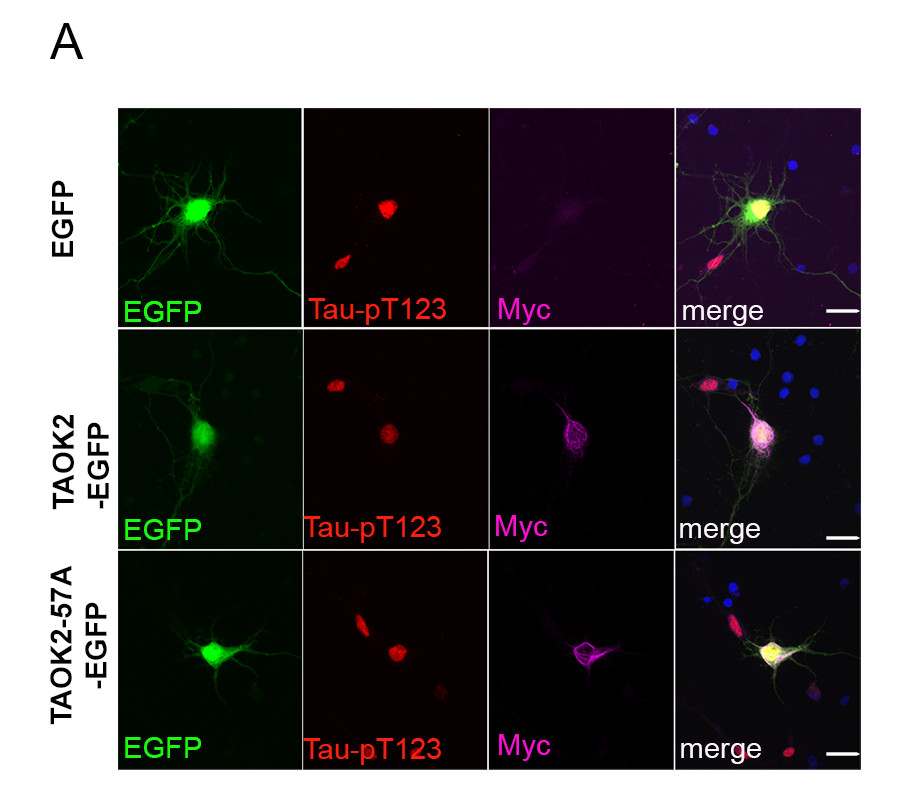

Supplement: Supplementary file 4 — Figure S3. Tau phosphorylated on T123 was present in the nuclei of rat primary neurons. A Neurons (7 DIV) were transfected with IRES-GFP, TAOK2-IRES-GFP or TAOK2 K57A-IRES-GFP, fixed and immunostained with antibodies to detect tau-pT123 (red) and Myc (magenta). Nuclei were counterstained with DAPI (blue). Representative images are shown. Scale bar = 10 μm. (TIF 409 kb) [file 40478_2018_539_MOESM4_ESM.tif]

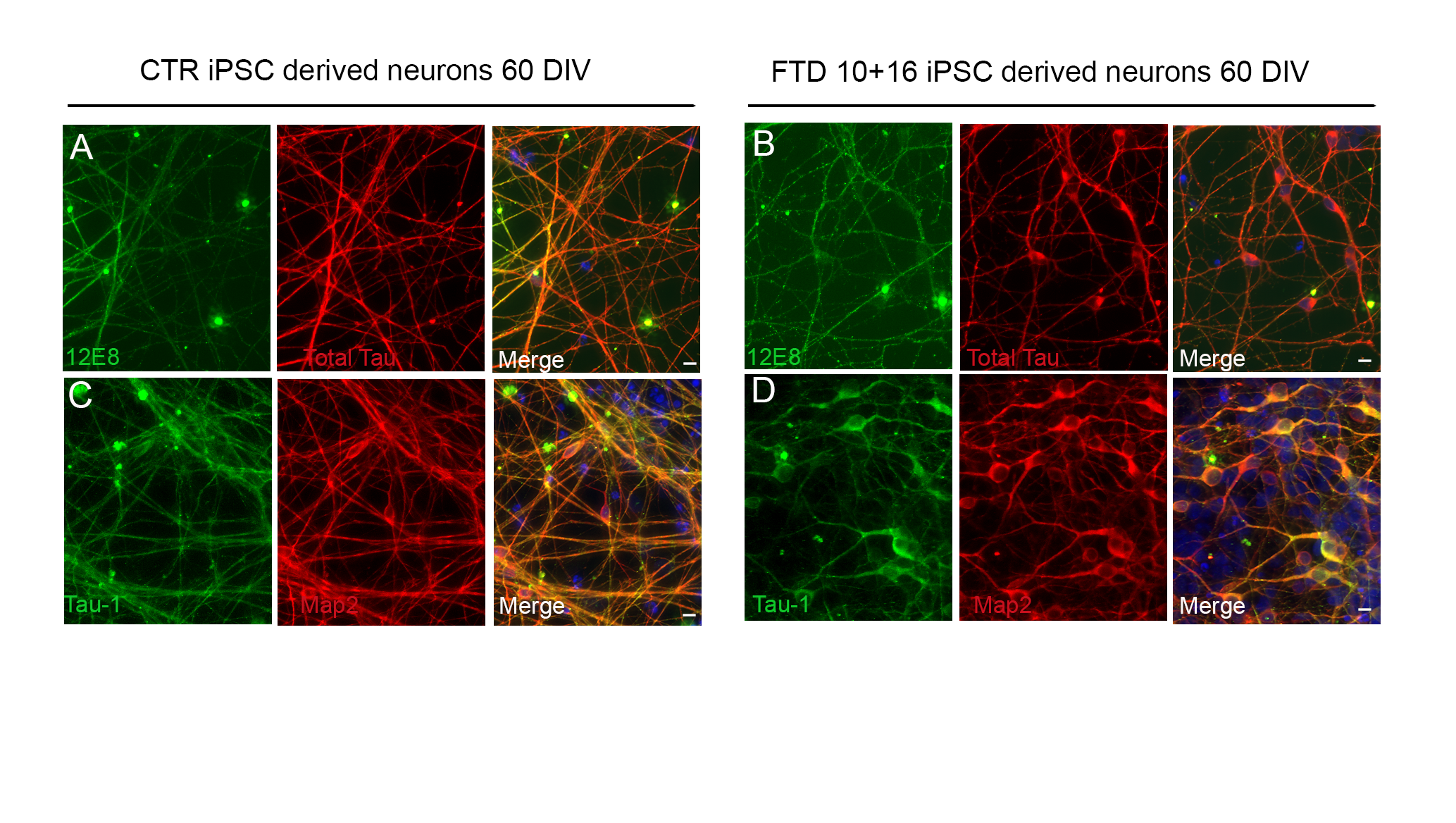

Supplement: Supplementary file 5 — Figure S4 FTLD and control iPSC-derived neurons expressed phosphorylated tau. A-D. FTLD (10 + 16 MAPT mutation) and control iPSC-derived neurons (60 DIV) were fixed and immmunostained with antibodies to detect total tau (red, A-B), tau-12E8 (green, A-B), tau-1 (green, C-D) and Map2 (red, C-D). Nuclei were counterstained with DAPI (blue). Representative images are shown. Scale bar = 10 μm. (TIF 4365 kb) [file 40478_2018_539_MOESM5_ESM.tif]
